# LncRNA ANRIL promotes HR repair through regulating PARP1 expression by sponging miR-7-5p in lung cancer

**DOI:** 10.1186/s12885-023-10593-z

**Published:** 2023-02-08

**Authors:** Zhipeng Du, Fangxiao Zhang, Lei Liu, Hui Shen, Tingting Liu, Jing Jin, Nanxi Yu, Zhijie Wan, Hang Wang, Xuguang Hu, Yuanyuan Chen, Jianming Cai

**Affiliations:** 1 School of Public Health and Management, Wenzhou Medical University, University Town, Wenzhou, Zhejiang P. R. China; 2 Department of Oncology, General Hospital of Central Theater Command of Chinese People’s Liberation Army, Wuhan, Hubei P. R. China; 3 Department of Radiation Medicine, Faculty of Naval Medicine, Naval Medical University, Shanghai, P. R. China; 4 Department of Occupational and Environment Health, Xiangya School of Public Health, Central South University, Changsha, Hunan P. R. China; 5 Department of Gastrointestinal Surgery, Changhai Hospital, Shanghai, P. R. China; 6 South Zhejiang Institute of Radiation Medicine and Nuclear Technology, Wenzhou, Zhejiang P. R. China

**Keywords:** Lung cancer, ANRIL, Radiation resistance, Homologous recombination repair, miR-7-5p, PARP1

## Abstract

**Background:**

Radiotherapy is an important treatment for lung cancer, mainly by triggering DNA double-strand breaks to induce cell death. Blocking DNA damage repair can increase the radiosensitivity of tumor cells. Recent studies have identified long noncoding RNAs as key regulators in DNA damage repair. The lncRNA ANRIL was previously shown to be involved in homologous recombination (HR) repair, but its specific mechanism has not been fully elucidated.

**Methods:**

The downstream interacting miRNAs of ANRIL were predicted according to miRanda software. Fluorescence quantitative PCR was used to detect the expression levels of ANRIL and candidate miRNAs. Clone formation experiment and cell viability assays detect cell viability after ionizing radiation. Apoptosis assay was used to detect the apoptosis of cells after 8 h of ionizing radiation. Western blot analysis and immunofluorescence assays verified the protein expression levels of the downstream target molecule PARP1 of miR-7-5p and key molecules in the HR pathway. Fluorescent reporter gene experiments were used to verify the interaction between ANRIL and miR-7-5p and between miR-7-5p and PARP1.

**Results:**

Bioinformatics analysis and qPCR validation suggested that miR-7-5p might be a downstream molecule of ANRIL. The expression of miR-7-5p was up-regulated after knockdown of ANRIL, and the expression of miR-7-5p was down-regulated after overexpression of ANRIL. Meanwhile, there was a negative correlation between ANRIL and miR-7-5p expression changes before and after ionizing radiation. The luciferase reporter gene assay confirmed the existence of ANRIL binding site with miR-7-5p, and found that transfection of miR-7-5p inhibitor can reduce the radiation sensitivity of ANRIL-KD cells. A downstream target molecule of miR-7-5p related to HR repair, PARP1, was screened through website prediction. Subsequently, it was confirmed by Western blot and luciferase reporter assays that miR-7-5p could down-regulate the expression of PARP1, and there was a miR-7-5p binding site on the 3'UTR of PARP1 mRNA. This suggests that ANRIL may act as a competitive endogenous RNA to bind miR-7-5p and upregulate the expression of PARP1. Western blot and immunofluorescence staining were used to detect the expression changes of HR repair factors in ANRIL-KD cells after ionizing radiation, and it was found that knockdown of ANRIL can inhibit the expression of PARP1, BRCA1 and Rad51, hinder radiation-induced HR repair, and eventually result in resensitizing ANRIL-KD cells to ionizing radiation.

**Conclusions:**

Our findings provide evidence that ANRIL targets the miR-7-5p/PARP1 axis to exert its regulatory effect on HR repair, suggesting that altering ANRIL expression may be a promising strategy to overcome radiation resistance.

**Supplementary Information:**

The online version contains supplementary material available at 10.1186/s12885-023-10593-z.

## Background

Lung cancer is the leading cause of cancer deaths worldwide due to its high morbidity and mortality [1]. It is generally believed that the occurrence of lung cancer is the result of a combination of factors, such as genetics, environment, food and lifestyle, among which DNA damage is thought to play some important roles [2]. Systemic treatment of lung cancer includes classical surgery, standard chemoradiotherapy, and monotherapy, and a combination of these methods is often recommended in clinical practice. Because many lung cancer patients are diagnosed with advanced cancer, they miss the best time for surgical intervention. At this time, radiotherapy and chemotherapy have become an important treatment method for patients with unresectable lung cancer [3]. Radiation therapy and platinum-based chemotherapy play a key role in therapy by disrupting DNA integrity and inducing tumor cell death. Even so, the 5-year survival rate for lung cancer has not improved significantly, remaining below 15% [4]. Clinical resistance to radiotherapy is considered a major obstacle to the treatment of human lung cancer. The main factor causing this phenomenon is the enhancement of tumor cells' ability to repair DNA damage induced by radiation [5]. Therefore, the effect of DNA damage repair ability on tumor radiosensitivity has attracted extensive clinical attention. The most serious type of DNA damage is DNA double-strand breaks (DSBs) [6], which, if not repaired in time, can lead to chromosomal aberrations, genomic instability, cancer, or cell death [7]. DSB repair is mainly carried out by two repair pathways: non-homologous end joining (NHEJ) and homologous recombination (HR) [8]. Blocking these repair pathways may lead to increased sensitivity to radiotherapy and chemotherapy.

The finding of long non-coding RNA (lncRNA) transcripts from genomics regions is one of the most unexpected findings of the genomics era. LncRNAs are a group of RNA transcripts over 200 nt in length that do not encode proteins but are involved in various forms of gene expression regulation [9]. Continuing evidence suggests that lncRNAs are involved in the development and progression of nearly all types of cancer [10], including effects on DNA damage repair capacity [11]. The lncRNA ANRIL is located in the 9q21.3 region of chromosome and was originally discovered and named by Pasmant et al. [12]. ANRIL has been found to be overexpressed as an oncogene in several different malignancies, such as liver [13], lung, [14] and esophageal cancers [15]. Both chemotherapy and radiotherapy can cause DNA damage in tumors. Studies have shown that the expression of ANRIL is related to the sensitivity of tumors to cisplatin. For example, MIAO demonstrated that ANRIL can affect ovarian cancer sensitivity to cisplatin by modulating the let-7a/HMGA2 axis [16]. WANG found that ANRIL could enhance the chemosensitivity of nasopharyngeal carcinoma cells to cisplatin [17]. Our previous study revealed that ANRIL can inhibit the radiosensitivity of lung cancer cells by promoting HR repair [18]. The molecular mechanism is that ANRIL mediates the recruitment of ATR protein to DNA breakage sites and maintains the stability of ATR protein [18]. Several studies have found that ANRIL can act as an adsorption sponge for miRNA, thereby regulating cell activity. For example, ZHANG et al. found that ANRIL can sponge miR-125a-3p and then regulate the expression of FGFR1 to promote the progression of HNSCC [19]. WANG demonstrated that ANRIL could activate HMGB1-induced autophagy by targeting miR-181a [20]. We therefore hypothesized whether ANRIL could also affect HR repair through a mechanism of competition for endogenous RNA (ceRNA). Following this line of thought, we found that ANRIL could regulate the miR-7-5p/PARP1 axis in the cytoplasm through website prediction and experimental verification, thereby affecting the radiosensitivity of lung cancer cells.

Therefore, we aimed to investigate whether ANRIL could modulate the HR repair pathway through the miR-7-5p/PARP1 axis in lung cancer cells, thereby altering the radiosensitivity of lung cancer cells. To provide evidence for finding a new strategy to alleviate the radiation resistance of lung cancer.

## Methods

### Cells and treatment

The normal epithelial cell of lung (BEAS-2B) and human lung cancer cells (A549, H1299, H460 and H1975) were all purchased from the American Type Culture Collection (ATCC, Manassas, VA, USA). A549, H460 and H1975 cells were maintained in DMEM medium with 10% fetal bovine serum (04–001-1ACS, Biological Industries, Israel), 50 U/mL penicillin, and 50 ug/mL streptomycin (Cat.15140–122, Gibco, USA) and cultured in a 37℃, 5% CO_2_ humidified chamber. BEAS-2B and H1299 cells were maintained in RPMI 1640 medium with the same supplement. Cells were treated with ionizing radiation to induce DNA damages.

### Cytoplasmic and nuclear RNA isolation assay

The total, cytoplasm and nuclear RNA were isolated using a Cytoplasmic and Nuclear RNA Purification Kit according to the manufacturer’s instruction (Norgen Biotek, Canada). U6 acted as nuclear internal control, and GAPDH acted as cytoplasmic internal control. Data normalization was performed against total RNA: % of Input = 100 X (2 ^[Ct total RNA – Ct fraction RNA]^).

### MiRanda predicts ANRIL-binding miRNAs

The target relationship between miRNA and lncRNA were predicted by using software miRanda v3.3a, with the parameter as follows: S ≥ 140, ΔG ≤  − 10 kcal/mol and demand strict 5' seed pairing, where S refers to the single-residue-pair match scores in the matching region; ΔG is the free energy of duplex formation.

### TargetScan, miRDB and Starbase predict target genes that interact with miR-7-5p

We use TargetScan, miRDB and Starbase biological information website to predict target molecules interacting with mir-7-5P. Select the target molecules with higher scores by setting the screening conditions. TargetScan: Sort by Cumulative Weight Context +  + Score, and then select the target molecules ranked in the top 200; MiRDB: Sort by Target Score, select the target gene ranked in the top two hundred; Starbase: Program Number >  = 2, and Program contains miRanda. The intersection of target molecules predicted by these three websites was used for subsequent analysis.

### Lentiviral vector construction and transfection

ANRIL shRNA plasmids and ANRIL overexpressing plasmid were constructed by Biolink BioTECH. (Shanghai, China) [18]. Sequence of sh-ANRIL: 5’-AAAUCCAGAACCCUCUGACAUUUGC-3’. The oligonucleotide was synthesized and cloned in a lentiviral vector: pLenR-GPH. Then, lentivirus stocks were generated by co-transfecting 293 T cells with the lentiviral constructs. Virus supernatants were collected at 48 h and 72 h after post-transfection, filtered, and concentrated by ultracentrifugation. H1299 or H460 cells were infected with the harvested recombinant virus. Virus-infected cells were plated in 6-well plates at 2X10^5^ cells per well, and screened in complete medium containing puromycin (2 μg/ml) to obtain stable transfected cell lines.Stable puromycin-resistant cell pools were established and confirmed with RT-PCR assay.

### MiRNA and siRNA transfection

The miR-7-5p mimic and inhibitor were synthesized by RiboBio (Guangzhou, China). The sequences of the synthetic oligonucleotides were as follows: miR-7-5p mimic sense: 5'-UGGAAGACUAGUGAUUUUGUUGUU-3', antisense: 5'-CAACAAAAUCACUAGUCUUCCAUU-3'; mimic NC sense: 5'-UUCUCCGAACGUGUCACGUTT-3', antisense: 5'-ACGUGACACGUUCGGAGAATT-3'; miR- 7-5p inhibitor: 5'-AACAACAAAAUCACUAGUCUUCCA-3'; inhibitor NC: 5'-CAGUACUUUUGUGUAGUACAA-3'. The PARP1 siRNA was synthesized by GenePharma (Shanghai, China), siRNA-PARP1 sense: 5'- CGACCUGAUCUGGAACAUCAA-3'; Negative control siRNA (NC-siRNA) sense: 5'- UUCUCCGAACGUGUCACGUTT-3'. These synthetic oligonucleotides were transiently transfected into H1299 or H460 cells by riboFECT™ CP (RiboBio, China) reagent according to the manufacturer’s protocol.

### Clone formation experiment

Clonal survival assays were used to assess the sensitivity of cells to ionizing radiation. Cells were counted and seeded into 6-well plates, and cells were subjected to DNA damage by ionizing radiation. Different irradiation doses correspond to different cell densities, 8 Gy is 2400 cells per well, 4 Gy is 1200 cells per well. Cells in 6-well plates were fixed with paraformaldehyde for 15 min after 10–14 days in the incubator and then stained with crystal violet for 15 min. The mean value ± SD for three independent experiments was determined.

### Cell viability assay

H1299-shANRIL, H460-ANRIL-OE cells or cells transfected with miR-7-5p inhibitor or siRNA were seeded in a 96-well plate at a density of 1000 cells per well, and 100 ul of medium was added to each well, and the next day was treated with ionizing radiation. Cell proliferation was detected using the Cell Counting Kit-8 (CCK-8, Dojindo Laboratories, Kumamoto, Japan) according to the manufacturer's instructions. Briefly, at the specified time point, 10ul of CCK-8 solution was added to each well, incubate at 37 °C for 4 h, and measure the absorbance value at 450 nm with a plate reader (BioTek, Vermont, USA) to determine the cell proliferation rate.

### Apoptosis assay

At 24 h after irradiation, cell apoptosis was detected by using an Annexin V-FITC and PI apoptosis detection kit according to the manufacturer’s instructions (Yeasen, Shanghai, China). FITC-Annexin V and PI dyes were added to the sample tubes and incubated in the dark for 10 min, and then the apoptosis of cells was analyzed by Cytoflex flow cytometer (Beckman, USA).

### Western blot analysis

The total protein from cells was extracted using RIPA lysis buffer. The protein lysates were separated by 10% SDS-PAGE and electrophoretically transferred to PVDF membranes (Millipore, USA). The membranes were incubated with primary antibodies at 4℃ overnight, followed by incubation with secondary antibodies how long. The signals were detected using an ECL system (Thermo Fisher Scientific, USA). The intensity of the protein fragments was quantified with Image J software. Anti-PARP1 antibody (1:1000, ab32138, Abcam), anti-BRCA1 antibody (1:1000, ab191042, Abcam), anti-Rad51 antibody (1:2000, ab133534, Abcam) and anti-GAPDH antibody (1:2000, ab8245, Abcam) were used as the primary antibodies for the detection of specific proteins.

### Immunofluorescence analysis

We used an immunofluorescence assay to detect BRCA1 and Rad51 foci. Briefly, cells concentration were seeded on 22 × 22mm^2^ cover glasses in 6 well plates. After ionizing radiation, cells were fixed in 4% paraformaldehyde for 20 min and permeabilized in 0.5% Triton X-100 on ice for 15 min. After blockage, cells were stained with anti-BRCA1 antibody (1:200, ab16780, Abcam) or anti-Rad51 antibody (1:250, ab133534, Abcam) and then with the secondary antibody. Cellular images were obtained using a confocal microscope (Nikon Eclipse Ti-SR, Japan). Image Pro Plus (Media Cybernetics) were used to count the foci per cell, and at least 100 cells per group were counted.

### RNA extraction and Realtime PCR RT and qPCR

The total RNA was extracted from cells using TRIzol reagent (Invitrogen). The mRNA was then reverse transcribed using the Primer-Script One Step RT-PCR Kit (TaKaRa, Japan) and the miRNA was reversely transcribed using the miRNA First strand cDNA Synthesis Kit (Sangon Biotech, China). The cDNA templates of ANRIL and miR-7-5p were amplified by real-time PCR using SYBR Premix Dimmer Eraser Kit (TaKaRa, Japan) and MicroRNAs qPCR Kit (Sangon Biotech, China), respectively. Gene expression in each sample was normalized to GAPDH expression. The primer sequences used were as follows: for GAPDH, 5’-CAGGAGGCATTGCTGATGAT-3’ (forward) and 5’-GAAGGCTGGGGCTCATTT-3’ (reverse); for ANRIL, 5’-CAACATCCACCACTGGATCTTAACA-3' (forward) and 5’-ATCATTCTCCTCAAATTACAGAG-3’ (reverse); for PARP1, 5’-AGCTTCGTATCCCCAATGAGATACA-3’ (forward) and 5’-TTTCCATCAAACATGGGCGAC-3’ (reverse). The forward primer for miR-7-5p was (5’-CGGTGGAAGACTAGTGATTTTGTTG-3’) and the reverse primer for U6 was (5’-CTCGCTTCGGCAGCACA-3’). The universal reverse primer was (5’-AACGCTTCACGAATTTGCGT-3’). The relative expression fold change of mRNA and miRNA was calculated by 2^−△△Ct^ method.

### Luciferase reporter assay

PmirGLO, pmirGLO-ANRIL-wt or pmirGLO-ANRIL-mut were co-transfected with miRNA-7-5p mimics or miRNA NC into 293 T cells in a 6-well dish using Lipofectamine 3000 reagent (Invitrogen, USA) according to the manufacturer’s instructions, respectively. PmirGLO, pmirGLO-PARP1-wt or pmirGLO-PARP1-mut were transfected with miRNA-7-5p mimics or miRNA NC into 293 T cells using Lipofectamine 3000 reagent (Invitrogen, USA) according to the manufacturer’s instructions, respectively. 48 h after transfection, luciferase activity was tested by Dual-Glo^@^ Luciferase Assay System (Promega, USA). Rinella luciferase activity was normalized to the corresponding firefly luciferase activity.

### MiRNA pulldown

Two 10 cm dishes were cultured with about 2 × 10^7^ cells. Biotin-labeled miRNA probes (experimental group) and negative control probes (negative control group) were transfected, respectively. After 48 h of culture, the cells were washed twice with precooled PBS. MiRNA pulldown Kit (BersinBio, Guangzhou, China) was used for the miRNA pulldown test. Magnetic bead sealing, cell lysis, hybridization and incubation, elution and precipitation of RNA were carried out in sequence according to the instruction. After the pulldown test was completed, qRT-PCR was used for follow-up detection.

### Nude mice transplanted with human lung cancer cells and ionizing radiation treatments

The whole protocols were approved by the Ethics Committee of Naval Medical University, China. Female BALB/c-nu nude mice of 4-week age were subcutaneously injected with H1299-shANRIL/NC. When the Average Tumor Diameter was at Least 500mm^3^, we carried out local ionizing radiation on the mouse's transplant tumor area with a dose of 20 Gy. After irradiation, the volume of subcutaneous tumors is detected every three days. The tumor volume was calculated according to the following formula (L*W^2^)/2 after measuring the length (L) and width (W) with calipers. After the experiment, the mice were killed and their tumors were taken out for mass weighing. The excised tumors were fixed at 4℃ in paraformaldehyde and embedded in paraffin. Tumors sections were used for immunohistochemistry analyses.

### Immunohistochemical staining

In this study, the tumor tissues in each group were paraffin-embedded and sliced, and then the tissues were stained with the Ki67 immunohistochemistry kit and Tunel analysis kit referring to the manufacturer’s method. Hematoxylin and eosin were used to stain paraffin sections of tumor tissues after deparaffinization and dehydration. Finally, observe the cell morphology under a microscope. Image-Pro Plus version 6.0 software assess the area and integrated option density (IOD), and then the mean density values were calculated, mean density = IOD/area.

### Statistical analysis

Data were expressed at the means ± standard error of mean. GraphPad Prism 8 software was applied for statistical analysis. For comparison among multiple groups, one-way ANOVA was performed. Student’s *t* test was used to compare between two groups. *p* < 0.05 was considered as significant difference. All experiments were performed at least 3 independent times.

## Results

### Identification of potential ANRIL-targeted miRNAs

**Fig. 1:**
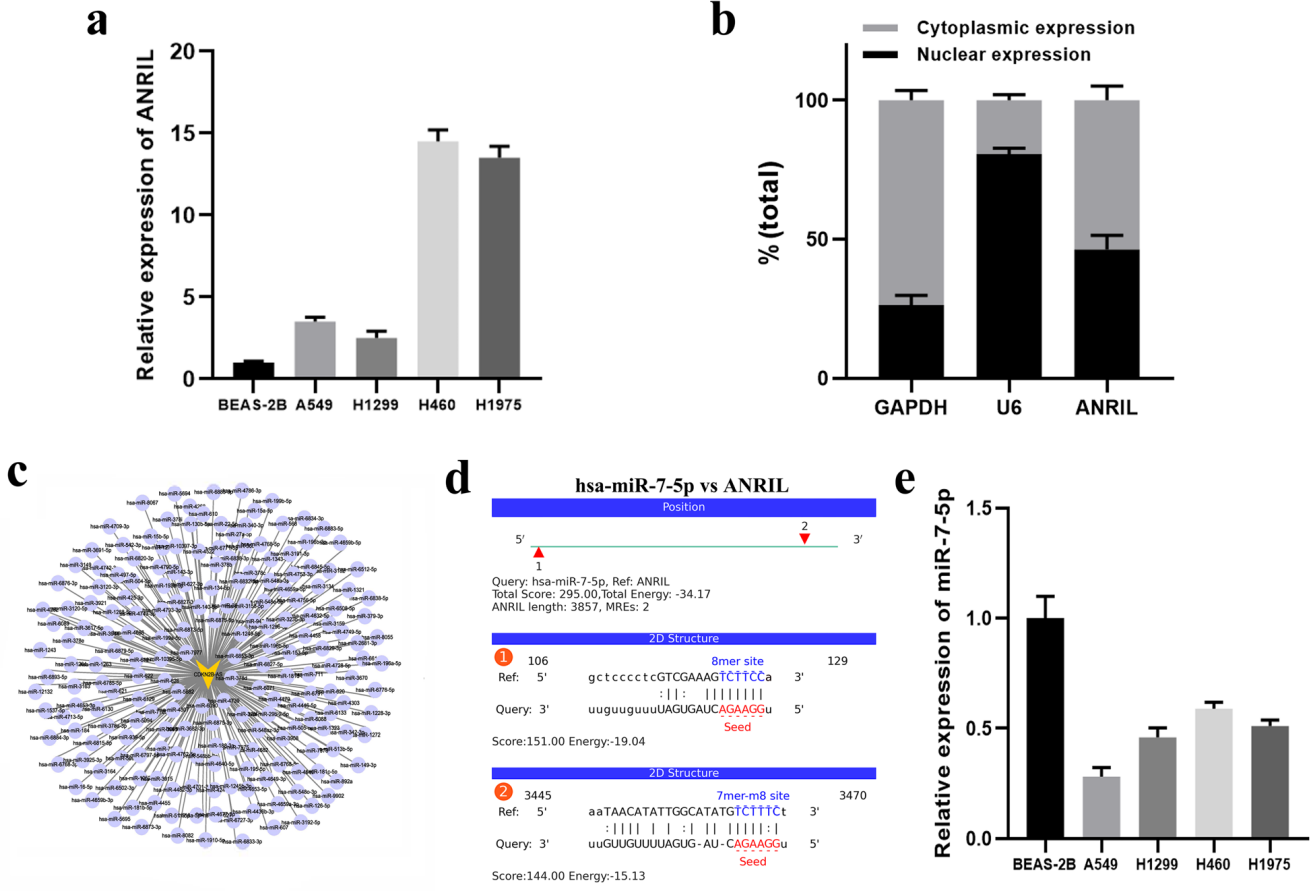
miRanda software predicts miRNAs targeting ANRIL. **a** ANRIL expression in human lung epithelial BEAS-2B cells and the human lung cancer cell lines A549, H1299, H460 and H1975. **b** qRT-PCR analysis of ANRIL expression in the nucleus and cytoplasm of H1299 cells. **a** A set of miRNAs that interact with ANRIL. **c-d** A schematic diagram of the interaction between miR-7-5p and ANRIL was drawn according to the prediction results of miRanda. **e** The expression levels of miR-7-5p in human lung epithelial BEAS-2B cells and the human lung cancer cell lines A549, H1299, H460 and H1975

Several studies have found that ANRIL is highly expressed in tumor cells and can act as a sponge to absorb miRNA, thereby regulating the biological characteristics of tumor cells. We detected the expression of ANRIL in various lung cancer cell lines and normal lung epithelial cells, and found that the expression level of ANRIL has significantly increased in lung cancer cells compared to normal lung epithelial cells (Fig. 1a).

We have previously shown that ANRIL can promote HR repair after ionizing radiation, so we wondered whether ANRIL could promote HR repair through a ceRNA mechanism. To test this hypothesis, we first examined expression levels and locations of ANRIL in H1299 cells. As shown in Fig. 1b, we found that ANRIL exists in large quantities in cytoplasm and nucleus, and the distribution ratio is similar. This suggest that ANRIL can play a key role as an endogenous sponge in cytoplasm. We used miRanda v3.3a software to predict ANRIL-binding miRNAs and obtained a set of miRNAs (Fig. 1c) (Supplementary Table 1). Sorted according to the total score, the top 25 miRNAs with the total score were selected for validation.

**Fig. 2:**
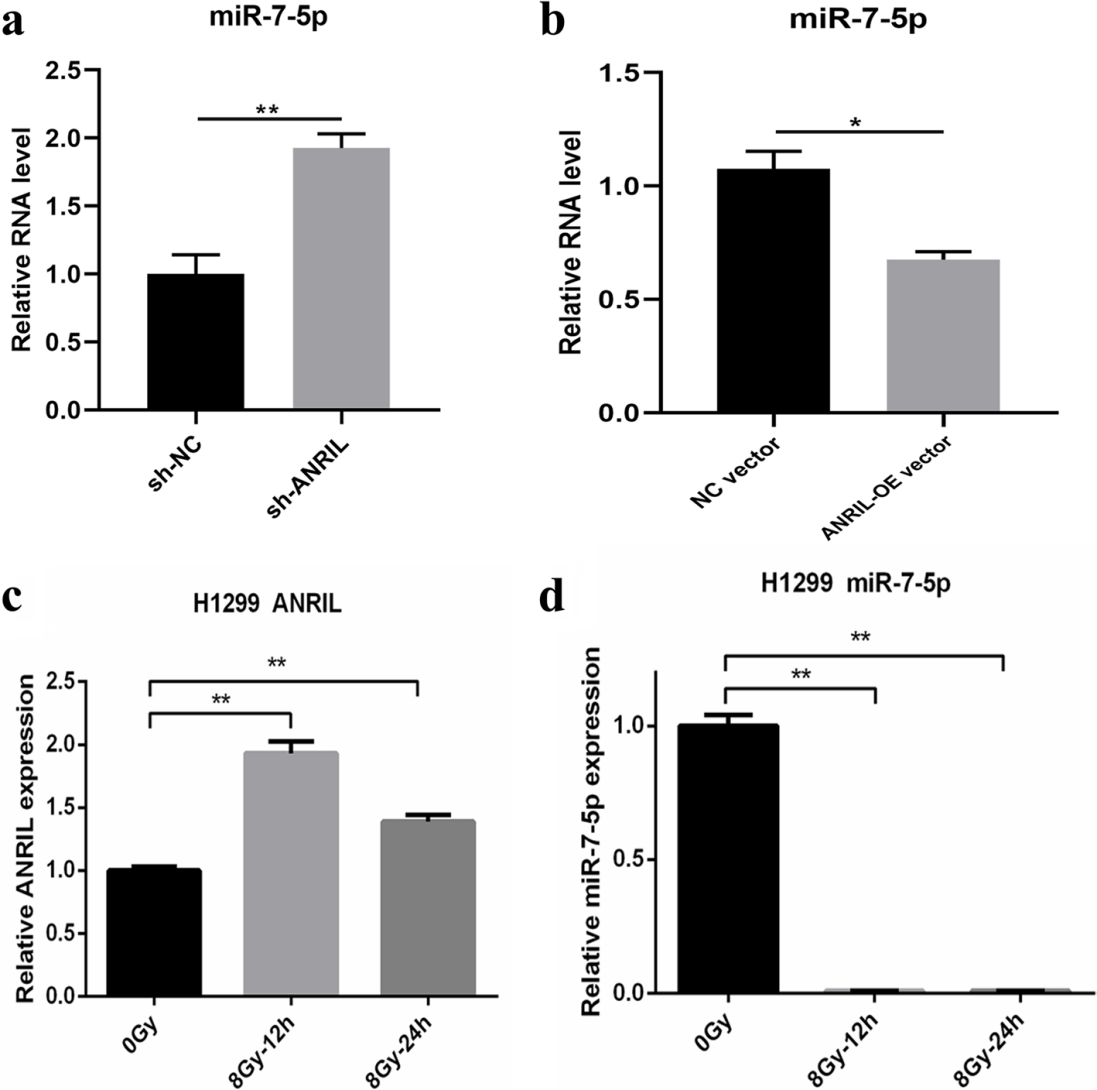
Identification of miR-7-5p as a target of ANRIL. **a-b** Relative expression of miR**-**7**-**5p in ANRIL shRNA or OE-transfected HA1299 or H460 cells. **c** Changes of ANRIL expression in H1299 cells before and after ionizing radiation. **d** Changes in the expression of miR-7-5p in H1299 cells before and after ionizing radiation

In our previous studies, we have established a knockdown cell line H1299-shANRIL in H1299 cells with high ANRIL expression and an overexpression cell line H460-ANRIL-OE in H460 cells with low ANRIL expression. Then the total 25 miRNAs were detected in the H1299-NC and ANRIL-KD cell line, it was found that the expression of 8 microRNAs was significantly increased, namely miR-7-5p, miR-15b-5p, miR-33b-5p, miR-122-5p, miR-125a-3p, miR-134-5p, miR-199a-5p, miR-497-5p (Supplementary Fig. 1). Then we further tested the expression of these eight microRNAs in H460 ANRIL-OE cell line and found that only the expression of miR-7-5p was significantly decreased (Fig. 2a-b). So far, we initially screened out a microRNA that may interact with ANRIL. We found that the binding score (total score) and thermodynamic stability (total energy) between ANRIL and miR-7-5p were high according to the prediction of miRanda software (Fig. 1d). We detected the expression of miR-7-5p in several lung cancer cells and normal lung epithelial cells, and found that the expression of miR-7-5p in normal lung epithelial cells was significantly higher than that in several lung cancer cells (Fig. 1e).

Previously, we found that the expression level of ANRIL increased after ionizing radiation, so we also detected the expression changes of miR-7-5p. We found that the expression level of miR-7-5p was significantly decreased after ionizing radiation, which was negatively related or correlated to the expression change of ANRIL (Fig. 2c-d). These data all indicate that ANRIL is a potential target molecule of miR-7-5p.

### ANRIL suppresses the radiosensitivity of lung cancer cells under ionizing radiation by targeting miR-7-5p

**Fig. 3:**
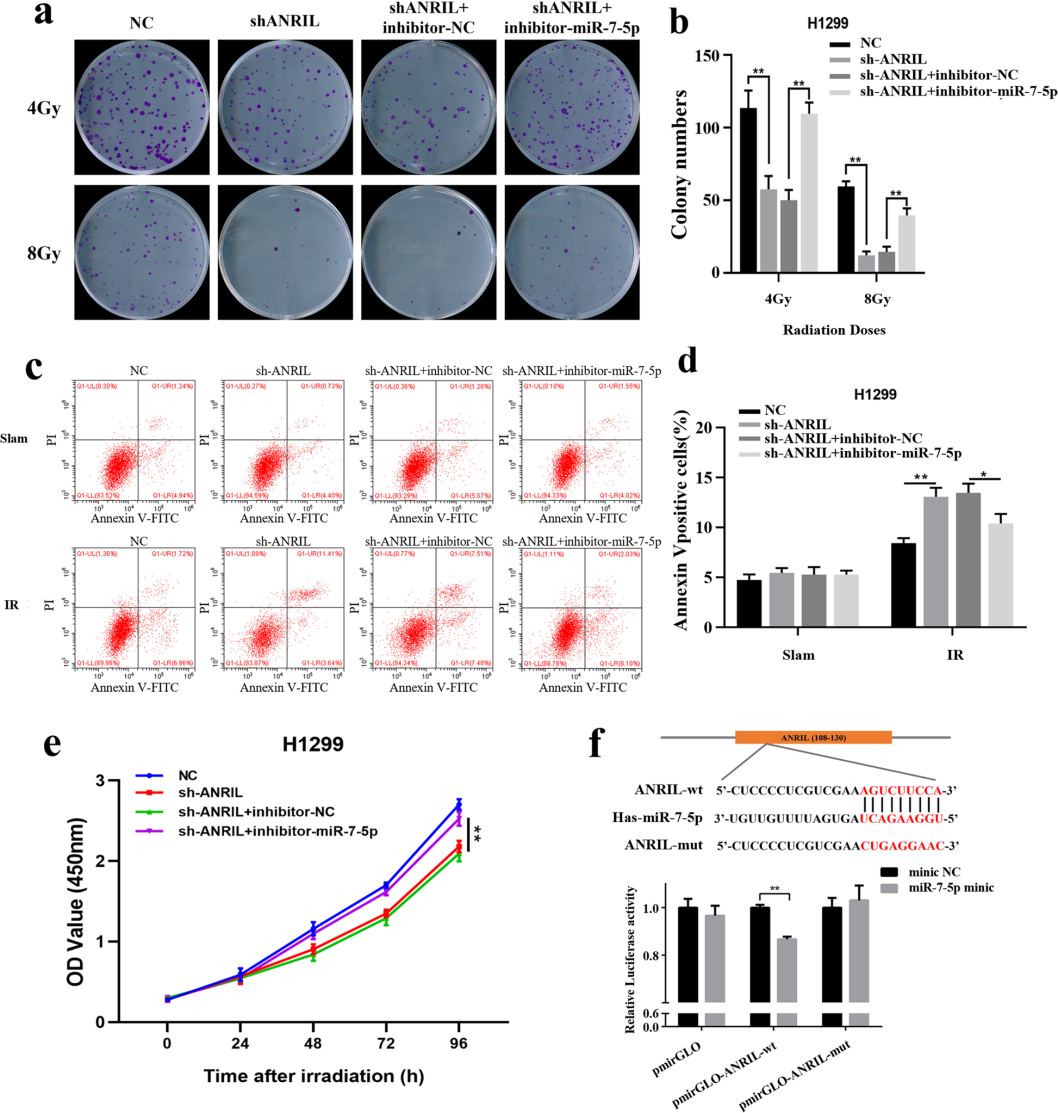
ANRIL inhibits radiosensitivity of lung cancer cells by targeting miR-7-5p. **a-b**, **e** Cell growth and viability after ionizing radiation were assayed in NC-, shANRIL-, shANRIL + inhibitor-NC- and shANRIL + inhibitor-miR-7-5p-transfected H1299 cells by CCK-8 assays and clone formation. **c-d** The apoptosis after ionizing radiation was detected in NC-, shANRIL-, shANRIL + inhibitor-NC- and shANRIL + inhibitor-miR-7-5p-transfected H1299 cells by flow cytometry. **f** ANRIL was mutated at the putative binding site. Luciferase activity in 293 T cells co-transfected with mimic-NC or mimic-miR-7-5p and luciferase reports containing NC, ANRIL-wt or ANRIL-mut transcripts. The results are presented as relative ratio of firefly luciferase activity to Renilla luciferase activity. *p < 0.05; **p < 0.01; ***p < 0.001

Our previous study demonstrated that knockdown of ANRIL in lung cancer cells can increase the radiosensitivity of the cells. Next, we transfected inhibitor-miR-7-5p in ANRIL knockdown H1299 cells, and the results of CCK8 assay (Fig. 3e) and clone formation assay (Fig. 3a-b) revealed that transfection of inhibitor-miR-7-5p significantly alleviated cell mortality after ionizing radiation. Similarly, apoptosis experiments showed that inhibition of miR-7-5p also reduced the increase in apoptosis levels caused by ANRIL knockdown (Fig. 3c-d).

**Fig. 4:**
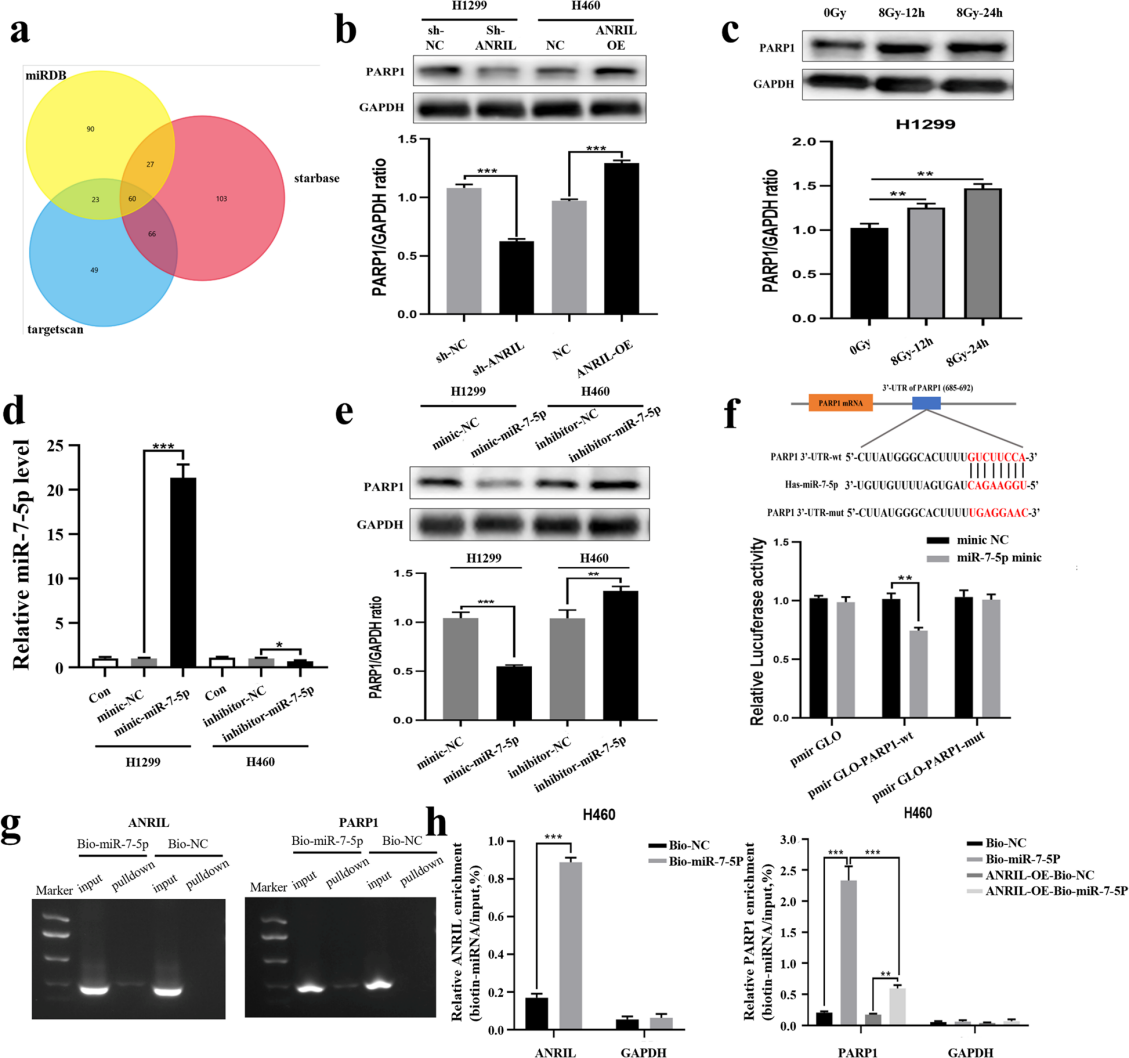
PARP1 was the target gene of miR-7-5p in lung cancer. **a** Venn diagram shows the target genes of miR-7-5p predicted by targetscan, miRDB and starbase bioinformatics website. **b** Effects of ANRIL on PARP1 expression levels in lung cancer cells. **c** Changes in protein expression of PARP1 before and after ionizing radiation irradiation. **d** Change of miR-7-5p expression levels in mimic-NC-, mimic-miR-125a-3p-, inhibitor-NC- and inhibitor-miR-7-5p-transfected H1299 and H460 cells. **e** Changes in PARP1 protein levels after transfection of mimic-miR-7-5p or inhibitor-miR-7-5p in lung cancer cells. **f** Mutations were made at the putative binding site of the PARP1 3'UTR region. Luciferase activity in 293 T cells co-transfected with mimic-NC or mimic-miR-7-5p and luciferase reports containing NC, PARP1-wt or PARP1-mut transcripts. The results are presented as relative ratio of firefly luciferase activity to Renilla luciferase activity. **g-h** Enrichment of ANRIL and PARP1 pulled down by biotin-miR-7-5p or negative control. Error bars represent the mean ± S.D. of triplicate experiment. *p < 0.05; **p < 0.01; ***p < 0.001

In view of the above results, we further confirmed the potential binding site of miR-7-5p on ANRIL by luciferase reporter gene assay. First, we constructed ANRIL-wt and ANRIL-mut luciferase reporter plasmids, which were co-transfected into 293 T cells with miR-7-5p mimic or NC, respectively. The results showed that only the pmirGLO-ANRIL-wt group significantly decreased the relative fluciferase activity when miR-7-5p was overexpressed, and there was no significant change in the pmirGLO-ANRIL-mut group and pmirGLO group. This indicates that miR-7-5p could directly bind to the predicted site in ANRIL (Fig. 3f). Further detection by the biotinase-coupled miRNA pulldown test showed that ANRIL can be pulled down by the miR-5-p biotin probe. Through qRT-PCR detection and calculation of the proportion of pulldown/input, this indicates that miR-7-5p can directly bind to ANRIL (Fig. 4g-h).

### PARP1 competitively binds miR-7-5p in cells

Next, we predicted the genes associated with miR-7-5p through targetscan, miRDB and starbase bioinformatics website. After selecting the intersection, there were a total of 60 genes (Fig. 4a) (Supplementary Table 2). Because we have proved that ANRIL is related to HR repair in the previous stage, we screened the genes with high scores and related to HR repair pathway from these candidate genes, and finally selected PARP1 as the target molecule of miR-7-5p for further verification. Studies have confirmed that PARP1 can bind to BRCA1, enter the nucleus, and promote the recruitment of RAD51, thereby promoting HR repair. Therefore, we detected the expression of PARP1 in ARNIL knockdown cells and overexpressed cells, and the results showed that PARP1 was positively correlated with ARNIL expression (Fig. 4b). Then we observed the expression changes of PARP1 in wild-type H1299 cells after ionizing radiation, and found that the expression level of PARP1 increased after ionizing radiation (Fig. 4c). Next, we found that the expression of PARP1 was decreased after transfection of mimic-miR-7-5p in H1299 cells and increased after transfection of inhibitor-miR-7-5p in H460 cells (Fig. 4d-e). All the above results suggest that PARP1 is likely to be a competitor of ANRIL binding to miR-7-5p. In order to verify this conjecture, we continued to verify it through dual luciferase reporter gene experiments. First, PARP1 3'UTR-wt and PARP1 3'UTR-mut luciferase reporter plasmids were constructed and co-transfected into 293 T cells with miR-188-5p mimic or NC, respectively. The results confirmed that PARP1 could directly bind to miR-7-5p at the predicted site (Fig. 4f). Further miRNA pulldown experiment showed that miR-7-5p could directly bind to PARP1. At the same time, after the overexpression of ANRIL in H460 cells, the amount of miR-7-5p binding PARP1 mRNA was reduced, with significant difference. This is consistent with our hypothesis that ANRIL can competitively bind miR-7-5p with PARP1 (Fig. 4g-h).

### ANRIL promotes homologous recombination repair by regulating miR-7-5p/PARP1/BRCA1/RAD51 signaling pathway

**Fig. 5:**
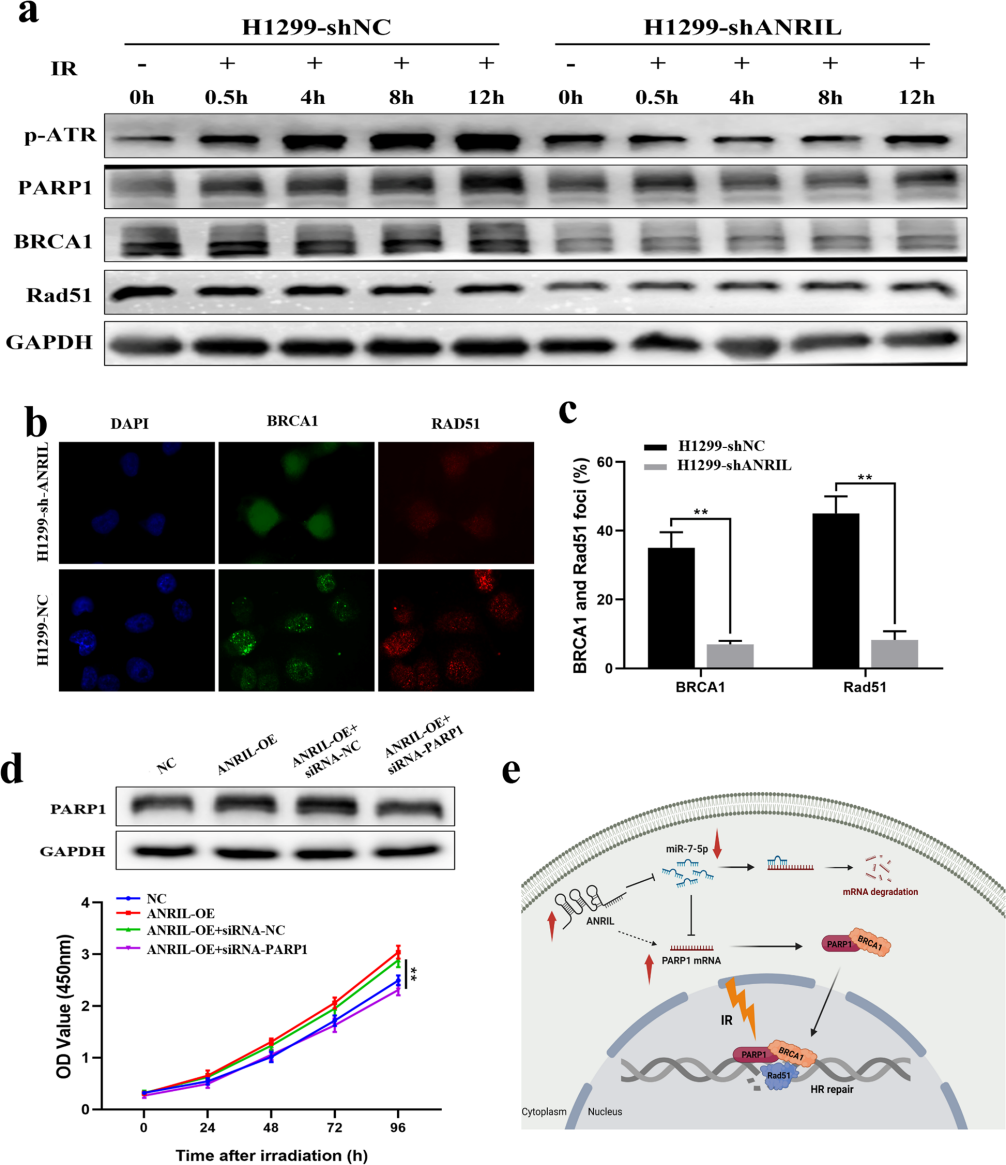
Promote of the HR repair pathway by ANRIL/miR-7-5p/PARP1 axis in lung cancer cells. **a** Western blot experiments detected the protein expression changes of PARP1, BRCA1 and RAD51 in ANRIL knockdown cells after ionizing radiation. **b-c** Immunofluorescence assays detected the foci recruitment of BRCA1 and Rad51 in ANRIL knockdown cells after ionizing radiation. **d** Cell growth after ionizing radiation were assayed in NC-, ANRIL-OE-, ANRIL-OE + siRNA-NC- and ANRIL-OE + siRNA-PARP1-transfected H460 cells by CCK-8 assays. **e** A working model of ANRIL promoting HR repair after ionizing radiation. ANRIL upregulation promotes HR repair after DNA damage by targeting the miR-7-5p/PARP1/BRCA1/RAD51 axis, conferring lung cancer cells resistant to radiotherapy. *p < 0.05; **p < 0.01

To verify that PARP1 is a downstream molecule of ANRIL in regulating cellular radiosensitivity, we performed rescue experiments. ANRIL-OE cells were transfected with siRNA-PARP1 and given 8 Gy ionizing radiation, and the CCK8 assay showed that the cell viability was inhibited (Fig. 5d). Studies have demonstrated that PARP1 can bind to BRCA1, enter the nucleus and promote the recruitment of RAD51, ultimately activating HR repair. Therefore, we detected the protein expression changes of PARP1, BRCA1 and RAD51 in ANRIL knockdown cells after ionizing radiation by western blot experiment, and the results showed that the expression of these proteins was inhibited after ANRIL knockdown (Fig. 5a). On the contrary, the expression of these molecules increased after ANRIL overexpression (Supplementary Fig. 2). Immunofluorescence assays further showed that knockdown of ANRIL affected the recruitment of BRCA1 and RAD51 foci (Fig. 5b-c), indicating that ANRIL could affect HR repair by regulating the PARP1/BRCA1/RAD51 pathway (Fig. 5e).

### The growth of transplanted tumor in nude mice

**Fig. 6:**
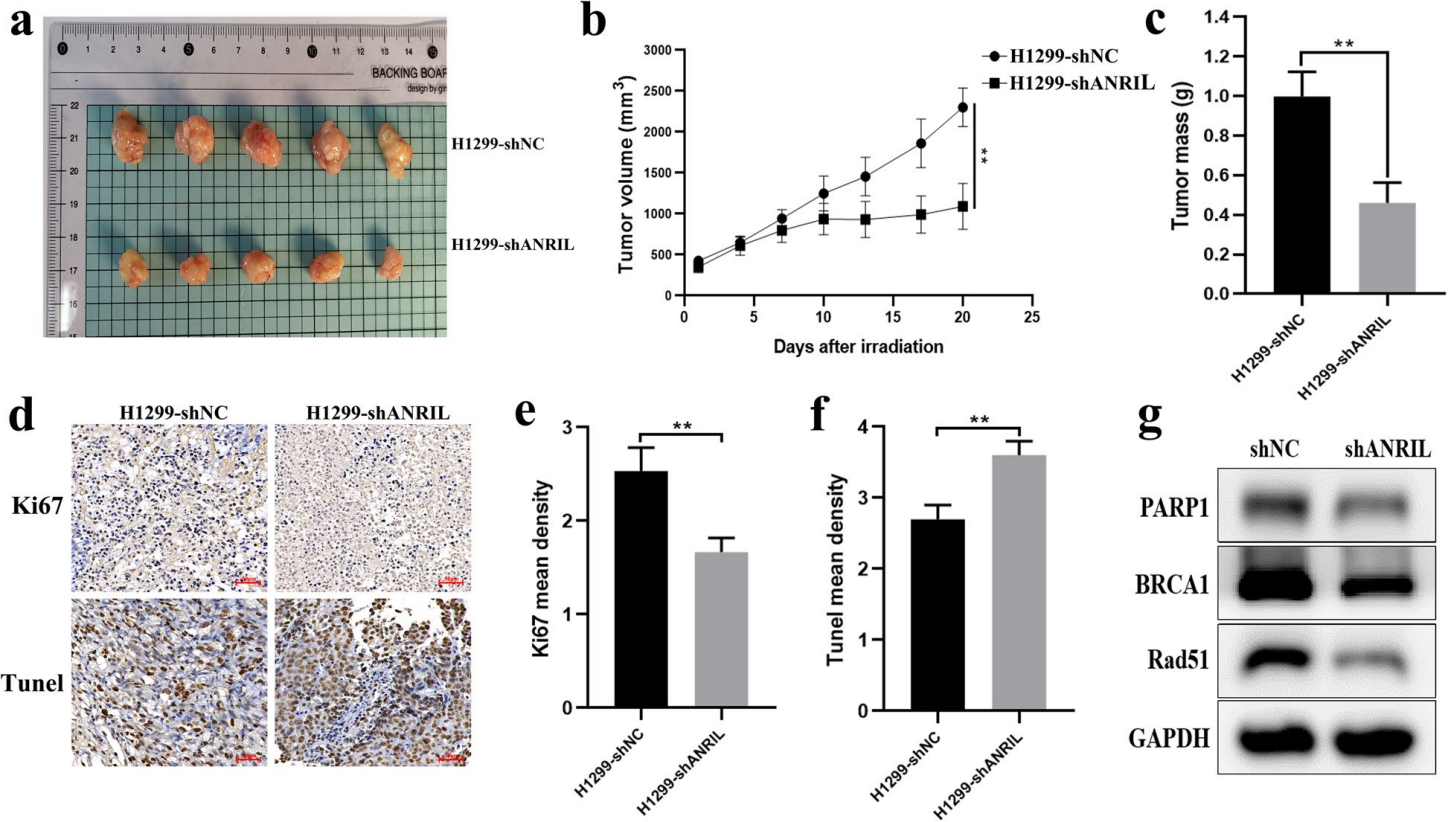
ANRIL shRNA increased radiosensitivity in xenograft nude mice. Tumor volume was measured every 3 days. At 24 days after the local ionizing radiation, the mice were killed and the tumors were stripped. **a** Photographs of tumors in nude mice. **b** The tumor growth curve and (**c**) tumor weight of transplantation tumor of nude mice. **d** Tumor section was carried out for immunohistochemistry of Ki67 and Tunel. **e**–**f** Respective mean density of Ki67 and Tunel in tumor by quantitative analysis. **g** The protein levels of a panel of core factors in HR signaling were reduced in shANRIL group

We attempted to demonstrate in vivo whether ANRIL can affect radiotherapy efficacy by targeting the miR-7-5p/PARP1 axis. We chose 4-week-old female nude mice to inject H1299-shNC and H1299-shANRIL cells subcutaneously. When the tumor grew to about 500mm^3^, ionizing radiation was applied to the transplanted tumor area. Observing the change of subcutaneous tumor in mice after irradiation over time, we found that the growth rate of tumor in shANRIL group was significantly slower than that in shNC group (Fig. 6a, b), and the weight of tumor in shANRIL group was also significantly lower than that in shNC group (Fig. 6c). The results of immunohistochemical staining showed that Ki67 representing tumor cell proliferation was significantly higher in shNC than shANRIL group (Fig. 6d). However, tunnel representing apoptosis was significantly less in shNC group than in shANRIL group (Fig. 6d). Western blot results showed similar results as in vitro experiments (Fig. 6e). These results indicate that ANRIL knockdown can promote the radiotherapy effect of tumor in vivo, and the mechanism is achieved by affecting the miR-7-5p/PARP1 axis. Overall, our findings indicated that ANRIL reduces Rad51 and BRCA1 expression by targeting miR-7-5p/PARP1, which ultimately leads to radiotherapy resistance in lung cancer cells.

## Discussion

Radiation therapy is a cancer treatment that uses high doses of ionizing radiation to induce cell death, primarily by triggering DNA double-strand breaks. Recently, long noncoding RNAs have been shown to affect ionizing radiation responses by modulating key signaling pathways, including DNA damage repair, apoptosis, glycolysis, cell cycle arrest, and autophagy [21]. LncRNA ANRIL is a recently frequently studied lncRNA molecule, which has been reported to promote the proliferation of a variety of solid tumors [22, 23]. The positioning of RNA determines its function. ANRIL ncRNA is mainly localized in the nucleus, and studies have shown that it can regulate gene transcription through chromatin remodeling. Our previous study also proved that ANRIL can combine with ATR to promote HR repair and reduce DNA damage [18]. Interestingly, several studies have found that ANRIL is also abundant in the cytoplasm. For example, in retinal cells, ANRIL was observed to localize to the perinuclear cytoplasmic fraction [24]. The presence of ANRIL was also observed in the cytoplasmic space of melanoma cells [25], suggesting that it is likely also involved in the post-transcriptional regulation of certain genes. Consistent with reports, ANRIL is associated with poor prognosis in cervical cancer, and in the cytoplasm, the ANRIL/miR-186 axis may promote carcinogenesis through the PI3K/Akt signaling pathway [23]. ANRIL overexpression promotes cell proliferation and migration through let-7a/TGF-β1/Smad cascade signaling [26]. We have demonstrated that ANRIL is distributed in the cytoplasm and nucleus of lung cancer cells through nucleocytoplasmic separation experiments. Therefore, we tried to explore whether ANRIL acts as a miRNA sponge in the cytoplasm to regulate radiosensitivity. After bioinformatics prediction and experimental validation, we identified miR-7-5p as a possible downstream molecule of ANRIL. The expression of ANRIL and miR-7-5p were negatively correlated after ionizing radiation. Knockdown of ANRIL significantly inhibited the radiation sensitivity of lung cancer cells, and the expression of miR-7-5p was increased. After overexpression of ANRIL, the expression of miR-7-5p was decreased. In rescue experiments, we found that a simulant of miR-7-5p could reverse the role of ANRIL in the regulation of radiosensitivity. Next, we demonstrated by dual-luciferase reporter experiments that ANRIL contains a binding site that can directly interact with miR-7-5p. These results suggest that ANRIL is involved in the regulation of radiosensitivity through miR-7-5p.

Previous studies have confirmed that miR-7-5p is a tumor suppressor microRNA and is involved in the process of various solid tumors, such as lung cancer [27], osteosarcoma [28], esophageal cancer [29], and liver cancer [30]. Decreased miR-7-5p can increase endogenous NOVA2 expression and inhibit tumor metastasis in non-small cell lung cancer [27]. miR-7-5p can also play a key role in radiation protection, and studies have shown that miR-7-5p can control radioresistance by producing ROS that leads to ferroptosis [31]. For clinically relevant radiation-resistant (CRR) cells, overexpression of miR-7-5p results in resistance to radiation [32]. These studies all indicate that miR-7-5p plays an important role in tumor development, especially in tumor response to radiation. We screened some candidate target molecules of miR-7-5p through several prediction methods. Since our previous article confirmed that ANRIL is involved in HR repair, we selected the molecule PARP1 most related to HR repair as miR-7-5p target molecule. PARP1 is considered to be a key protein involved in HR repair, and itself and the polymerization of ADP-ribose units it catalyzes are thought to rapidly recruit DNA damage proteins to DNA damage sites [33]. Ionizing radiation can cause DNA double-strand breaks (DSBs), and there are two main pathways for DSB repair: nonhomologous end joining (NHEJ) and homologous recombination (HR). PARP1 is a key determinant in the HR repair pathway, which is essential for maintaining genomic integrity and survival during radiotherapy. It has been found that miR-7-5p-mediated downregulation of PARP1 affects DNA homologous recombination repair and the resistance of lung cancer cells to doxorubicin [34]. Consistent with their study, we confirmed that transfection of miR-7-5p mimic could inhibit the expression of PARP1, and the expression of PARP1 was increased after transfection of inhibitor. Compared with wild-type lung cancer cells, knockdown of ANRIL decreased the expression of PARPA1, while overexpression of ANRIL increased the expression of PARP1. These results indicate that ANRIL may indirectly affect the expression of PARP1 through miR-7-5p, and the mechanism is endogenous competition for miR-7-5p. The dual-luciferase reporter gene assay further demonstrated that miR-7-5p can also directly interact with the 3'UTR of PARP1.

PARP1 contains the binding domain of BRCA1, which is an important interacting molecule of PARP1. Previous studies have demonstrated that PARP1 is critical for BRCA1 recruitment to DSB sites, and depletion of PARP1 results in a decrease in BRCA1 foci. The PARP1-BRCA1 complex further regulates DSB repair, including enabling Rad51 to bind to single-stranded DNA and guiding strand exchange [35]. Consistent with this observation, our results demonstrated that ANRIL promoted BRCA1 and Rad51 expression and foci recruitment following ionizing radiation. Our findings suggest that ANRIL can mediate HR repair through the miR-7-5p/PARP1/BRCA1/Rad51 signaling pathway and enhance DNA damage repair capacity.

## Conclusion

This study first elucidated the relationship between the ANRIL/miR-7-5p/PARP1 axis and HR repair in radiation resistance. Further studies showed that PARP1-mediated HR activity was regulated by ANRIL. Considering the indispensability of PARP1 in HR repair, our study demonstrates that ANRIL can serve as a potential biomarker of radioresistance and possibly a novel therapeutic target for overcoming radioresistance.

## Supplementary Information

**Additional file 1:** **Supplementary Table 1.** miRanda v3.3a software predicts ANRIL-binding miRNAs.

**Additional file 2:** **Supplementary Table 2.** Prediction of target genes for miR-7-5p by targetscan, miRDB and starbase bioinformatics website.

**Additional file 3:** **Supplementary Figure 1. **Detection of the expressionlevel of miRNAs by qRT-PCR. The expression of these miRNAs was detected in H1299-NC and ANRIL-KD cell lines. **Supplementary Figure 2. **Overexpression of ANRILactivates the PARP1/BRCA1/RAD51 pathway. Westernblot experiments detected the protein expression changes of PARP1, BRCA1 andRAD51 in ANRIL overexpression cells after ionizing radiation.

## Data Availability

All data generated or analyzed during this study are included in this published article and its supplementary information files.

## Abbreviations

HR: Homologous recombination
3’UTR: 3’-Untranslated region
DDR: DNA damage response
DSBs: Double-strand breaks

## References

[1] Siegel RL, Miller KD, Jemal A (2016). Cancer statistics, 2016. CA Cancer J Clin.

[2] Kastan MB (2008). DNA damage responses: mechanisms and roles in human disease: 2007 G.H.A. Clowes memorial award lecture. Mol Cancer Res..

[3] Ettinger DS, Wood DE, Akerley W, Bazhenova LA, Borghaei H, Camidge DR, Cheney RT, Chirieac LR, D'Amico TA, Dilling TJ (2016). NCCN guidelines insights: non-small cell lung cancer, version 4.2016. J Natl Compr Canc Netw.

[4] Spira A, Ettinger DS (2004). Multidisciplinary management of lung cancer. N Engl J Med.

[5] Willers H, Held KD (2006). Introduction to clinical radiation biology. Hematol Oncol Clin North Am.

[6] Panier S, Boulton SJ (2014). Double-strand break repair: 53BP1 comes into focus. Nat Rev Mol Cell Biol.

[7] Gao S, Feng S, Ning S, Liu J, Zhao H, Xu Y, Shang J, Li K, Li Q, Guo R (2018). An OB-fold complex controls the repair pathways for DNA double-strand breaks. Nat Commun.

[8] Fortini P, Ferretti C, Dogliotti E (2013). The response to DNA damage during differentiation: pathways and consequences. Mutat Res.

[9] Ntziachristos P, Abdel-Wahab O, Aifantis I (2016). Emerging concepts of epigenetic dysregulation in hematological malignancies. Nat Immunol.

[10] Evans JR, Feng FY, Chinnaiyan AM (2016). The bright side of dark matter: lncRNAs in cancer. J Clin Investig.

[11] Shaw A, Gullerova M (2021). Home and away: the role of non-coding RNA in Intracellular and Intercellular DNA damage response. Genes.

[12] Pasmant E, Sabbagh A, Vidaud M, Bièche I (2011). ANRIL, a long, noncoding RNA, is an unexpected major hotspot in GWAS. FASEB J.

[13] Li K, Zhao B, Wei D, Cui Y, Qian L, Wang W, Liu G (2020). Long non-coding RNA ANRIL enhances mitochondrial function of hepatocellular carcinoma by regulating the MiR-199a-5p/ARL2 axis. Environ Toxicol.

[14] Fisher L (2021). Retraction: Long noncoding RNA ANRIL knockdown increases sensitivity of non-small cell lung cancer to cisplatin by regulating the miR-656-3p/SOX4 axis. RSC Adv.

[15] Chen D, Zhang Z, Mao C, Zhou Y, Yu L, Yin Y, Wu S, Mou X, Zhu Y (2014). ANRIL inhibits p15(INK4b) through the TGFβ1 signaling pathway in human esophageal squamous cell carcinoma. Cell Immunol.

[16] Miao JT, Gao JH, Chen YQ, Chen H, Meng HY, Lou G (2019). LncRNA ANRIL affects the sensitivity of ovarian cancer to cisplatin via regulation of let-7a/HMGA2 axis. Biosci Rep.

[17] Wang Y, Cheng N, Luo J. Downregulation of lncRNA ANRIL represses tumorigenicity and enhances cisplatin-induced cytotoxicity via regulating microRNA let-7a in nasopharyngeal carcinoma. J Biochem Mol Toxicol. 2017;31(7):e21904. 10.1002/jbt.21904 28117929

[18] Liu L, Chen Y, Huang Y, Cao K, Liu T, Shen H, Cui J, Li B, Cai J, Gao F (2021). Long non-coding RNA ANRIL promotes homologous recombination-mediated DNA repair by maintaining ATR protein stability to enhance cancer resistance. Mol Cancer.

[19] Zhang LM, Ju HY, Wu YT, Guo W, Mao L, Ma HL, Xia WY, Hu JZ, Ren GX (2018). Long non-coding RNA ANRIL promotes tumorgenesis through regulation of FGFR1 expression by sponging miR-125a-3p in head and neck squamous cell carcinoma. Am J Cancer Res.

[20] Wang L, Bi R, Li L, Zhou K, Yin H (2021). lncRNA ANRIL aggravates the chemoresistance of pancreatic cancer cells to gemcitabine by targeting inhibition of miR-181a and targeting HMGB1-induced autophagy. Aging.

[21] Podralska M, Ciesielska S, Kluiver J, van den Berg A, Dzikiewicz-Krawczyk A, Slezak-Prochazka I (2020). Non-Coding RNAs in cancer radiosensitivity: MicroRNAs and lncRNAs as regulators of radiation-induced signaling pathways. Cancers.

[22] Wang ZW, Pan JJ, Hu JF, Zhang JQ, Huang L, Huang Y, Liao CY, Yang C, Chen ZW, Wang YD (2022). SRSF3-mediated regulation of N6-methyladenosine modification-related lncRNA ANRIL splicing promotes resistance of pancreatic cancer to gemcitabine. Cell Rep.

[23] Zhang JJ, Wang DD, Du CX, Wang Y (2018). Long Noncoding RNA ANRIL promotes cervical cancer development by acting as a sponge of miR-186. Oncol Res.

[24] Thomas AA, Feng B, Chakrabarti S (2017). ANRIL: a regulator of VEGF in diabetic retinopathy. Invest Ophthalmol Vis Sci.

[25] Sarkar D, Oghabian A, Bodiyabadu PK, Joseph WR, Leung EY, Finlay GL, Baguley BC, Askarian-Amiri ME (2017). Multiple isoforms of ANRIL in melanoma cells: structural complexity suggests variations in processing. Int J Mol Sci.

[26] Zhao B, Lu YL, Yang Y, Hu LB, Bai Y, Li RQ, Zhang GY, Li J, Bi CW, Yang LB (2018). Overexpression of lncRNA ANRIL promoted the proliferation and migration of prostate cancer cells via regulating let-7a/TGF-β1/ Smad signaling pathway. Cancer Biomark.

[27] Xiao H (2019). MiR-7-5p suppresses tumor metastasis of non-small cell lung cancer by targeting NOVA2. Cell Mol Biol Lett.

[28] Gu W, Chen P, Ren P, Wang Y, Li X, Gong M (2021). Downregulation of TAF9B by miR-7-5p Inhibits the Progression of Osteosarcoma. Onco Targets Ther.

[29] Shi W, Song J, Gao Z, Liu X, Wang W (2020). Downregulation of miR-7-5p Inhibits the tumorigenesis of esophagus cancer via targeting KLF4. Onco Targets Ther.

[30] Wang Y, Yang H, Zhang G, Luo C, Zhang S, Luo R, Deng B (2021). hsa-miR-7-5p suppresses proliferation, migration and promotes apoptosis in hepatocellular carcinoma cell lines by inhibiting SPC24 expression. Biochem Biophys Res Commun.

[31] Tomita K, Nagasawa T, Kuwahara Y, Torii S, Igarashi K, Roudkenar MH, Roushandeh AM, Kurimasa A, Sato T (2021). MiR-7–5p is involved in ferroptosis signaling and Radioresistance thru the generation of ROS in Radioresistant HeLa and SAS cell lines. Int J Mol Sci.

[32] Tomita K, Fukumoto M, Itoh K, Kuwahara Y, Igarashi K, Nagasawa T, Suzuki M, Kurimasa A, Sato T (2019). MiR-7-5p is a key factor that controls radioresistance via intracellular Fe(2+) content in clinically relevant radioresistant cells. Biochem Biophys Res Commun.

[33] Ray Chaudhuri A, Nussenzweig A (2017). The multifaceted roles of PARP1 in DNA repair and chromatin remodelling. Nat Rev Mol Cell Biol.

[34] Lai J, Yang H, Zhu Y, Ruan M, Huang Y, Zhang Q (2019). MiR-7-5p-mediated downregulation of PARP1 impacts DNA homologous recombination repair and resistance to doxorubicin in small cell lung cancer. BMC Cancer.

[35] Cruz-García A, López-Saavedra A, Huertas P (2014). BRCA1 accelerates CtIP-mediated DNA-end resection. Cell Rep.

