# Supplementary material for: LncRNA ANRIL promotes HR repair through regulating PARP1 expression by sponging miR-7-5p in lung cancer
Source: BMC Cancer. 2023 Feb 8;23:130. doi: 10.1186/s12885-023-10593-z (PMC9906921; doi:10.1186/s12885-023-10593-z)
Supplement: Supplementary file 3 — Additional file 3: Supplementary Figure 1. Detection of the expressionlevel of miRNAs by qRT-PCR. The expression of these miRNAs was detected in H1299-NC and ANRIL-KD cell lines. Supplementary Figure 2. Overexpression of ANRILactivates the PARP1/BRCA1/RAD51 pathway. Westernblot experiments detected the protein expression changes of PARP1, BRCA1 andRAD51 in ANRIL overexpression cells after ionizing radiation. [file 12885_2023_10593_MOESM3_ESM.docx]

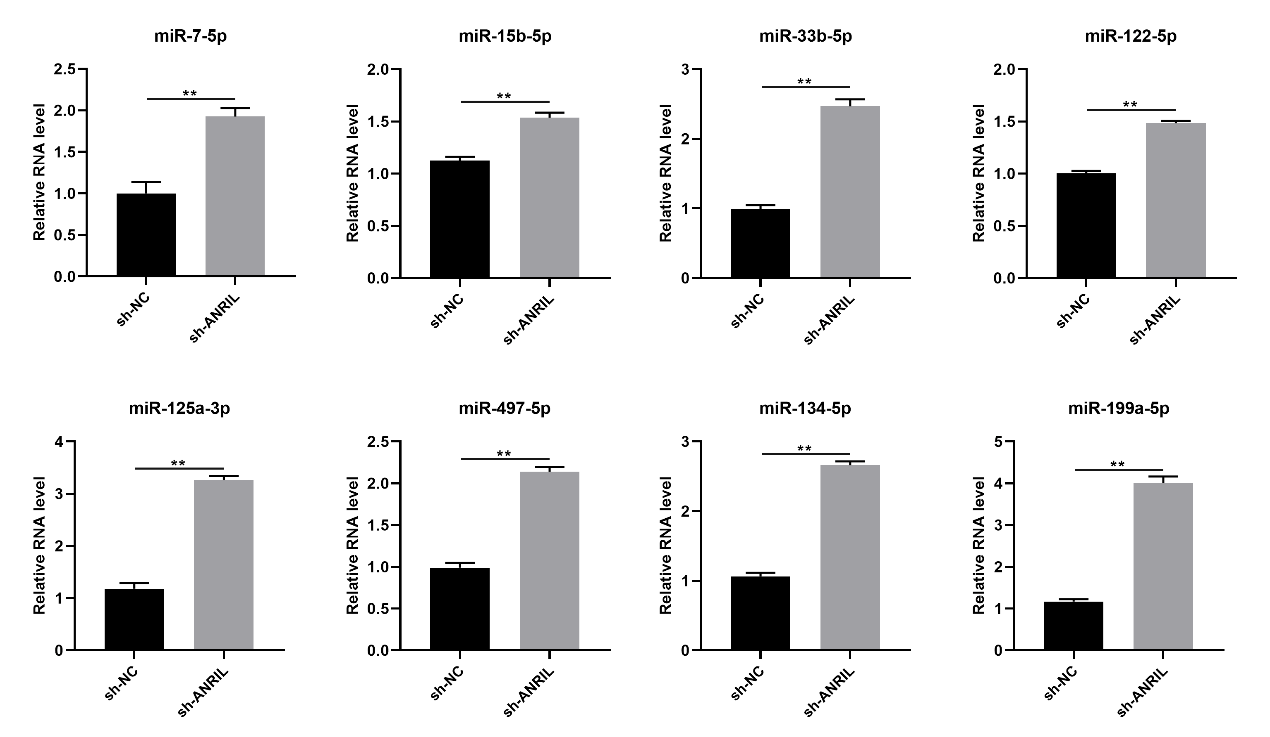


**Supplementary Figure 1.** Detection of the expression level of miRNAs by qRT-PCR. The expression of these miRNAs was detected in H1299-NC and ANRIL-KD cell lines.


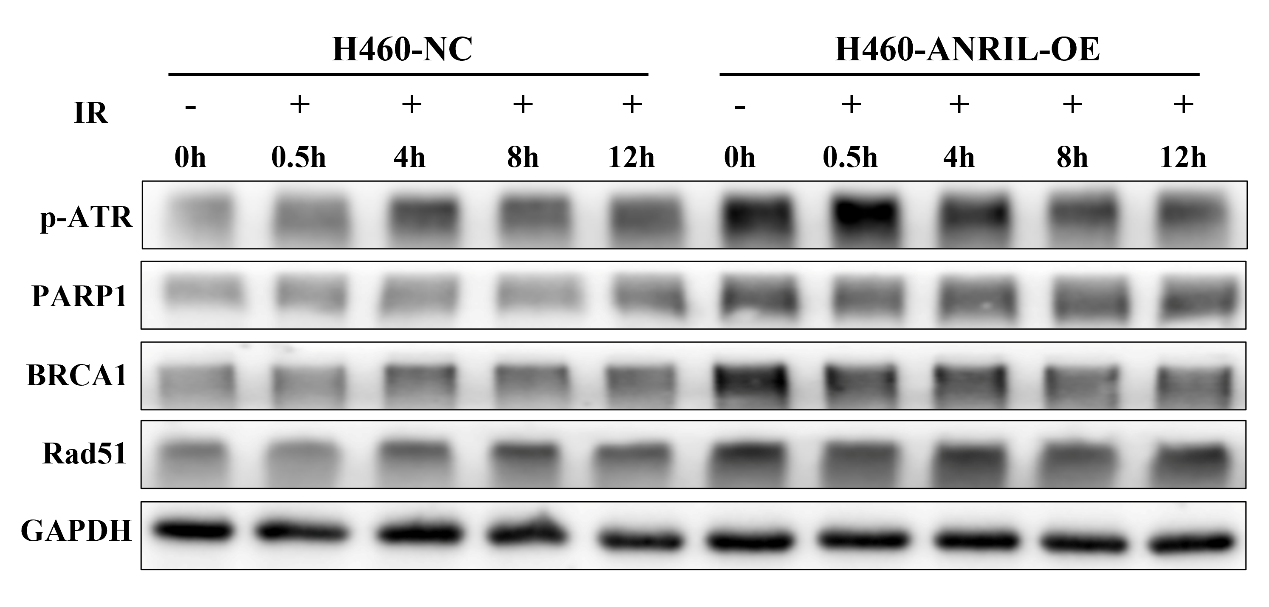


**Supplementary Figure 2.** Overexpression of ANRIL activates the PARP1/BRCA1/RAD51 pathway. Western blot experiments detected the protein expression changes of PARP1, BRCA1 and RAD51 in ANRIL overexpression cells after ionizing radiation.
